# The role of patients and carers in diffusing a health‐care innovation: A case study of “My Medication Passport”

**DOI:** 10.1111/hex.12893

**Published:** 2019-05-26

**Authors:** Susan Barber, Catherine French, Rachel Matthews, Derryn Lovett, Tom Rollinson, Fran Husson, Margaret Turley, Julie Reed

**Affiliations:** ^1^ NIHR Collaboration for Leadership in Applied Health Care and Research (CLAHRC) Northwest London, Chelsea & Westminster Hospital NHS Foundation Trust Department of Primary Care and Public Health, Imperial College London London UK; ^2^ University Hospitals of Derby and Burton NHS Foundation Trust Derby UK; ^3^ Guy's and Thomas' NHS Foundation Trust, St Thomas' Hospital London UK

**Keywords:** case study, co‐production, diffusion, health care, infrastructure support, innovation, patient's roles, self‐care

## Abstract

**Background:**

Patients are increasingly recognized as playing important roles in improving health services. Little is known about the mechanisms by which patients develop and diffuse local innovations in a complex health‐care system.

**Objective:**

To ascertain how diffusion of an innovation, My Medication Passport, occurred and roles played by patients in it.

**Design:**

Case study: quantitative mapping of innovation's diffusion and analysis of the routes and occupations of those through whom the innovation spread; documentary analysis; reflective assessment of patient's roles.

**Setting and participants:**

NHS Trusts, third sector organizations, patients and health‐care professionals.

**Interventions studied:**

Co‐produced action to raise awareness and influence use of the innovation; order database which enabled ease of access to the innovation.

**Main outcome measures:**

Geographical spread of innovation; occupations of individuals; types of organizations using the innovation.

**Results:**

The innovation spread from initial development and use in Northwest London across the UK and beyond. Key roles played by patients were as follows: co‐producer; advocate; relationship builder; relationship broker; planner; presenter; awareness raiser; trainer; networker. Patients identified and introduced potential audiences and users to MMP, using social, organizational, sectoral, lay and professional networks to do so. They organized a range of awareness‐raising and communication activities, monitored feedback, evaluated the impact and responded to new interest.

**Discussion and conclusions:**

The roles of patients in diffusing innovations are under‐recognized. Collaborative working between patients, carers and health‐care professionals in planning and progressing the use and supporting diffusion of the innovation was important. Principles described in this study are relevant to progressing other patient‐led ideas for innovative changes relating to health service development.

## 1 BACKGROUND

Patients, carers and members of the public are increasingly recognized as playing or as having the potential to play an important role in improving health services. This can include leading and developing innovative solutions to problems that matter to them. However, little is known about the roles played by patients, or the mechanisms by which patients can develop and diffuse local innovations in a complex health‐care system. Patient developed innovations rarely spread beyond their own personal use.

Oliveira et al^1^ suggest that when patients develop innovations, these are mostly used to help themselves, or those they care directly for, but are rarely shared more widely. They surveyed 500 patients who had rare diseases to measure how many had developed innovations to help themselves and/or support their own care. They found that 8% of the total number of patients who responded to the survey had developed innovative solutions for themselves that medical experts evaluated as novel. The majority of the innovations focused on increasing the patient's autonomy and quality of life. Lack of diffusion of patient innovation was due to significant barriers to doing so, including inventors' lack of time, skills and opportunities to develop their innovations, and lack of contact with communities who are likely to use the innovation. One of the conclusions of their study was that patients have the potential to transform health care but for that to happen they need to be supported and their input integrated into health‐care delivery.

Denis et al^2^ theorize that the dissemination of innovations is not necessarily a linear process which adheres to a plan, and that the process of diffusion can be discerned as an interaction between the innovation with its key characteristics and an adopting system, composed of actors with a set of values, interests and power dependencies. They argued that innovations and networks of supporting actors co‐evolve over time, and that the adoption and diffusion process is by nature dynamic. Their insight was that successful innovations tend to be adaptable to new contexts. The key principles that define the purpose and use of the innovation remain constant, regardless of context and type of user, and this, Denis et al^2^ conceptualized as the “hard core” of the innovation. Part of the reason for success is the adaptability of the innovation to new contexts, and this they conceptualized as the use of the innovation at the “soft periphery.”

Diffusion of innovation theory is now used across many disciplines and is generally traced to the 1950s and 1960s in the early work of Rogers.^3^, ^4^ Rogers' later revisions of that early work presented refinements to the definition(s) linked to the concept of communication and its role in diffusing innovations.^4^ He posited the view that communication which drives diffusion is not linear, and that a more helpful concept was that of convergence. Convergence occurs when communication that drives diffusion is characterized by individuals interacting together, sharing information, seeking information about an innovation and the extent to which the innovation meets a need, how, when and where it is tried, and their experience of it. This is referred to as communication among diffusion networks with information about the innovation sought from “near peers.”^5^

Greenhalgh et al^5^ reviewed the available literature and produced a conceptual model for considering the determinants of dissemination, implementation and diffusion of innovations in health service delivery and organization. Their review distinguished between dissemination (active and planned efforts to persuade target groups to adopt an innovation), implementation (active and planned efforts to mainstream an innovation within an organization) and diffusion of an innovation (passive spread beyond implementation).

Key limitations of diffusion theories have been noted^2^, ^4^, ^5^ among which are: it is extremely difficult to account for all the factors which might have played a part in diffusion, and that diffusion theories cannot account for all variables which are likely to be responsible. Most models of diffusion are presented as linear, when the reality is that there is often overlap between the different drivers of and motivations for it.

Patients as innovators in NHS or other health‐care settings are written about in policy, empirical and theoretical studies. Insights into patient roles in relation to developing, disseminating or diffusing innovations are rarely mentioned. Instead, the focus is on how patients can be, or are involved in health‐care planning, delivery, evaluation and research. Evidence of patients and carers influencing and successfully co‐producing service improvement is increasingly studied. Authors have noted that co‐production can enhance patient and clinician experience and patient and service outcomes, even though patients' experience and expertise are often not used to the fullest.^6^, ^7^, ^8^, ^9^, ^10^, ^11^ The positive impact that patient engagement and involvement in health care can have on outcomes, including improving patients' understanding of their health conditions, how these can be treated and managed, self‐management and peer‐to‐peer learning, is noted in empirical and policy‐focused studies.^12^, ^13^ Patients are increasingly taking on roles in setting research agendas, including suggesting research questions, becoming part of a research team and evaluating outcomes.^14^, ^15^, ^16^ Commentators, however, suggest that the potential for patients to participate in research continues to be underutilized.^17^, ^18^, ^19^ Patients' participation in self‐care and their potential to play important roles in enhancing the learning of their doctors is thought to be driving a scientific paradigm change in which the patient's voice is central to an emerging era of participatory medicine.^20^ In addition, guidelines for how, when and why to involve patients are increasingly published by patient‐led, third sector groups and used in NHS settings.^21^, ^22^ This literature provides a lens through which we can understand the landscape in which patients have played important roles.

Sheard et al^23^ considered the characteristics of award‐winning individuals working in health care who had been recognized as innovators influencing change in NHS settings. From interviews with 15 of them, four key themes emerged as significant: personal determination, the ability to broker relationships, navigating organizational culture to good advantage and the ability to use evidence to influence others. Barnett et al^24^ considered innovators' experiences of barriers and facilitators in implementation and diffusion of innovations and found that innovators themselves often go on to be important champions who are willing to lead efforts to achieve diffusion, and they drew attention to the significant influence of inter‐personal and inter‐organizational networks, the inner and outer context, and the evidence base for success.

My Medication Passport (MMP) is an innovation developed by and for patients and carers. Available as a hand‐held passport sized booklet, or as an app, it is used to record medicines taken, medication aids, allergies and changes to medicines. It can be used to record information about illnesses, vaccinations, hospital or GP appointments and screenings, who to contact in an emergency and anything else the user wishes to note.

Initially, the MMP innovation was used to support a service improvement project. The prototype for it came from a carer who had used a handwritten document previously. This was built‐on and co‐developed by patients, pharmacists and other health‐care professionals. It focused on prescribing and medicines review for elderly patients seen in acute care settings in five hospitals in Northwest London. It was initially intended to support patients when they were discharged from hospital. Transitions of care, for example when someone is discharged from hospital to community, pose risks for patients and can cause adverse events related to miscommunication and inadequate record sharing.^25^ There was no one comprehensive, universally available electronic patient health‐care record.^26^ Changes to medicines reviewed in hospitals were often not known about by GPs or community pharmacists and this caused confusion.^27^ An evaluation of MMP use demonstrated that it worked well as an aide‐memoire and to improve communication between patients, carers, doctors, pharmacists and other health‐care professionals. Subsequently, MMP was used by other types of user.^27^, ^28^

In 2013, the MMP innovation was launched outside the initial use of it in Northwest London and made available nationally and internationally. Patients and carers who had co‐led the development of the innovation as part of a health service improvement team subsequently joined a multidisciplinary steering group and undertook roles designed to diffuse it.

The National Institute for Health Research (NIHR) Collaboration for Leadership in Applied Health Research and Care Northwest London (NIHR CLAHRC NWL) provided a supportive context for patient and carer involvement in the development and dissemination of the MMP. It subsequently provided a platform to make the innovation widely available. In addition, it was responsive to new ideas and opportunities, recognizing the need for and supporting innovation in complex health‐care systems.^29^, ^30^ Methodologies shaped by NIHR CLAHRC NWL progressed iterative testing and development of the innovation, encouraging patients and carers to work as part of a service improvement team. This can be seen as part of a larger picture in which a policy context had supported greater attention being paid to enhancing patient involvement in health service improvement.^31^, ^32^

This study explores how diffusion of the MMP innovation occurred and the roles that patients and carers played in it. In addition, it explores what factors facilitated their role in the diffusion of the innovation and identifies the processes and mechanisms by which MMP was diffused.

## 2 DESIGN

Using a case study approach^33^, we conducted a mixed‐methods evaluation: quantitative analysis of an electronic database used to store data relating to orders received by the provider of the MMP innovation; and qualitative analysis of documents linked to patient/carer roles in diffusion of MMP.

This study was approved by the Health Research Authority (IRAS 188851). We had explicit permission from those quoted in the Results section of the study to use those quotations. People who placed orders shared the following information: name; occupation; email; address; post‐code. When they placed their order, they indicated their willingness yes, or no, to being contacted again for research purposes.

### 2.1 Data collection

We accessed a database used to record how many orders for the MMP innovation were fulfilled, where in the country those orders were sent, and who they were sent to. Between 5 April 2013 and 31 September 2017, 164 000 were ordered.

We accessed n = 51 documents. These were related to the diffusion of the innovation after it had been developed, tested and implemented on five health‐care sites in Northwest London. See Table 1.

**Table 1:** Documents and other sources accessed

Type of document	Number
Records of steering group meetings to make MMP available nationally	12
Grey literature/newsletters containing articles about MMP	11
Presentations used in awareness‐raising events and workshops	9
Posters shown at conferences	6
Vignettes showing records of how MMP was used/diffused—collected from records including email correspondence, websites, first‐hand reports by users	3
Peer‐reviewed publications about the early use and evaluation of MMP	3
Planning documents relating to making MMP available outside of its original use in Northwest London	2
Plan about how to make MMP available nationally	2
Record of communication events, which included monitoring notes written by patients and health‐care professionals (detailing 74 outreach activities)	1 plan
Order database	1
Training materials for pharmacists	1
Video used in training and presentations	1
Total	51

### 2.2 Data analysis

From the electronic order database, we analysed how many MMPs were ordered and the extent of diffusion from 5 April 2013 to 30 September 2017. An analysis of the database using an Excel spreadsheet enabled us to understand who had ordered the innovation, their occupation and where in the UK they were based. The database was analysed using geographical information system software, and post‐code analysis conducted, which was mapped to a visual output (Figures 2 and 3).

**Figure 1:**
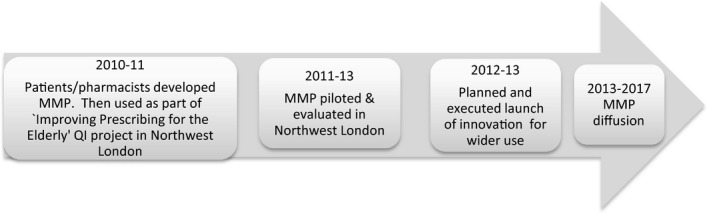
My medication passport: innovation diffusion timeline. My Medication Passport (MMP) was co‐developed, designed and tested by patients, carers, pharmacists and other healthcare professionals in 2010‐11. It was disseminated by five hospitals in Northwest London and its use piloted 2011‐13. Its use was evaluated. A steering group assembled in 2012‐13 with the aim of making MMP widely available to anyone who would like to make use of it. From 2013‐17 MMP diffused, aided by an ordering service

From the 51 documents, we compiled a timeline giving an overview of the development through to the diffusion of MMP. See Figure 1. From documents relating to the work of the steering group, which was convened to make the MMP innovation available to anyone who would like to use it, the roles of patients in planning and raising awareness about the transition from dissemination to diffusion of the MMP were noted. Roles played by patients are summarized in Table 2, types of events they co‐produced in Table 3, and audiences who attended the events in Table 4.

**Table 2:** Roles played by patients in communication events

*Roles*
Advocate for the use of the innovation
Awareness raiser
Distributor of innovation
Networker
Planner and co‐producer
Presenter
Relationship builder and relationship broker
Trainer

**Table 3:** Dissemination and diffusion events (indicative examples)

*Meetings*
Local pharmaceutical committee
Metropolitan police force and prison officers
National patient safety meeting
National third sector organization
Regional pharmacy group
*Networking*
Charities and third sector organizations
National renal network
NIHR CLAHRC NWL research network
Specialist pharmacists networking event—leading to adoption by hospital outside NWL
*Publicity*
Ethnic Minority Health Network
Faith‐based organization
Libraries
National pharmacy chain
Presentations at conferences
Radio interview
*Workshops*
Dentists' and dental nurses' workshop to raise awareness about MMP
NHS Social Care MMP workshops and advocacy linked to support for people with mental health issues
Police force training and workshops to support people who take multiple medicines and who lived with learning disabilities, mental health needs, were homeless.
Prison officers training and workshops to support people who take multiple medicines and who lived with learning disabilities, mental health needs, were homeless.
Pharmacist managers training and workshops

**Table 4:** Dissemination and diffusion event attendees

Community services
Librarians
NHS primary and secondary care clinicians and allied health professionals
People living with a learning disability and/or mental health need
Pharmacists
Police
Prison officers
Public
Public health
Researchers
Third sector

We purposively identified three tracer cases which we present as vignettes 1, 2 and 3. The vignettes were chosen to allow for an exploration of the types of roles of patients and carers who were not involved in the Northwest London‐based steering group or the initial pilot sites in Northwest London. The vignettes provided an insight into how people who ordered the innovation found out about it, why they ordered it or recommended its use, and in what context. Vignettes 1 and 2 were sourced from correspondence with users of the innovation and from observations of its mention in published and grey literature; vignette 3 was sourced from observations of its mention by organizations which provide information and support to users and health‐care organizations.

### 2.3 Limitations

This study had four key limitations. First, it was impossible to collect comprehensive data about all the roles played by all the patients and health‐care professionals involved in the diffusion process. The study focuses mostly on patient's and carer's roles because there is a gap in the literature about this. Pharmacists and other health‐care professionals played similar roles. A study of their specific roles in diffusing MMP also merits attention but a full account of this is outside the scope of this study. Second, it was not possible to form a comprehensive understanding of how all individuals and groups who ordered MMP might have influenced the diffusion of the innovation outside of Northwest London. Third, our literature review excluded how patients may shape the diffusion of innovations using technological means, such as apps or Internet. Fourth, no model of network analysis was used to analyse the networking described in the paper.

## 3 RESULTS

### 3.1 Geographical spread and diffusion of the MMP innovation

The timeline presented in Figure 1 starts with the development of the MMP innovation which was initially part of a service improvement project in Northwest London. The timeline shows the trajectory of progress to diffusion.

Figure 1 presents a linear timeline. However, our evidence demonstrates the process was emergent, with action, especially in the diffusion period characterized as spread: geographically, and via individuals, networks and organizations, in a non‐linear manner.

**Figure 2:**
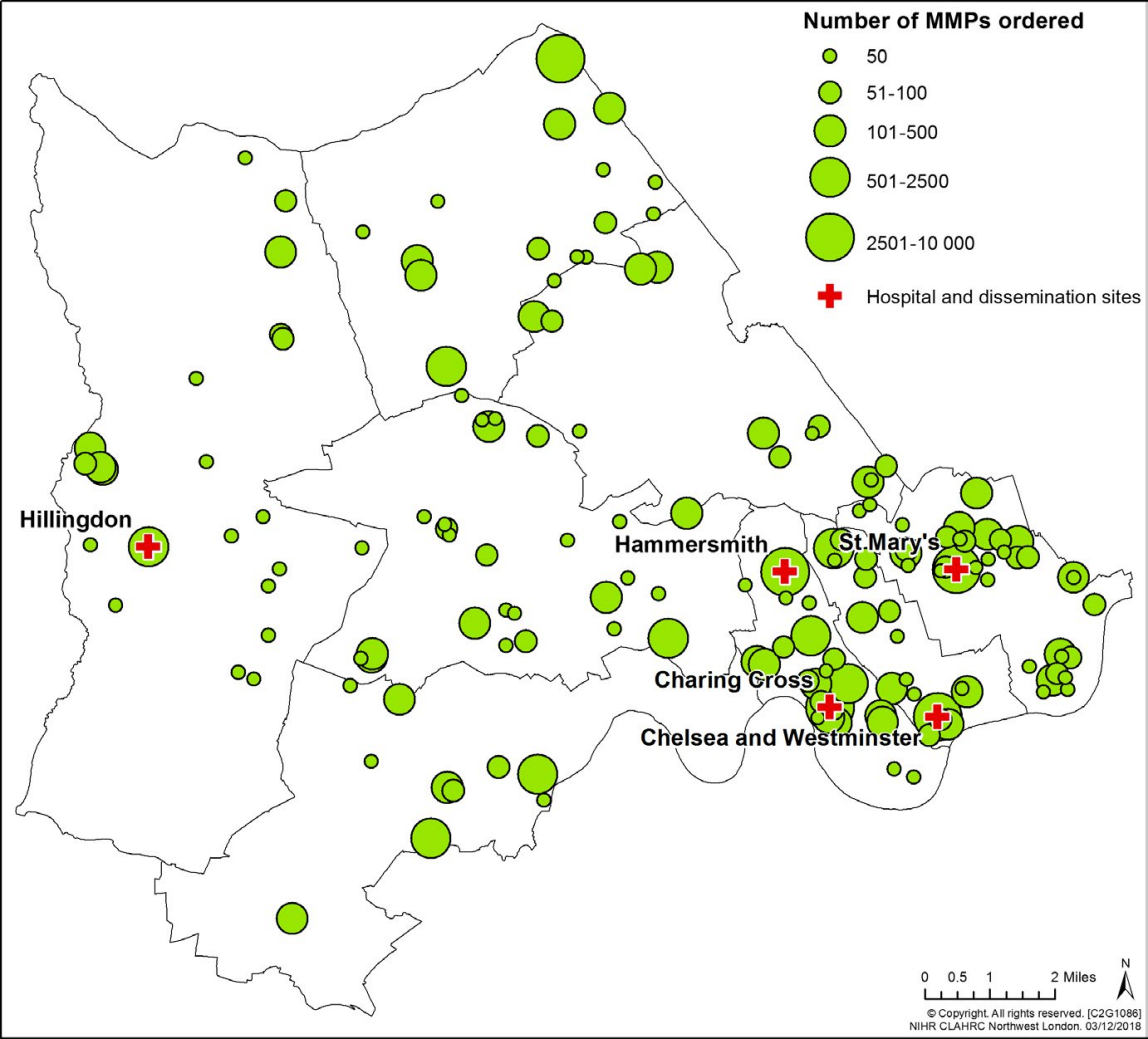
Diffusion of MMP in Northwest London. North West London boroughs showing hospital/dissemination sites involved in MMP service improvement project and number of MMP orders by location between 5th April 2013 and 30th September 2017

**Figure 3:**
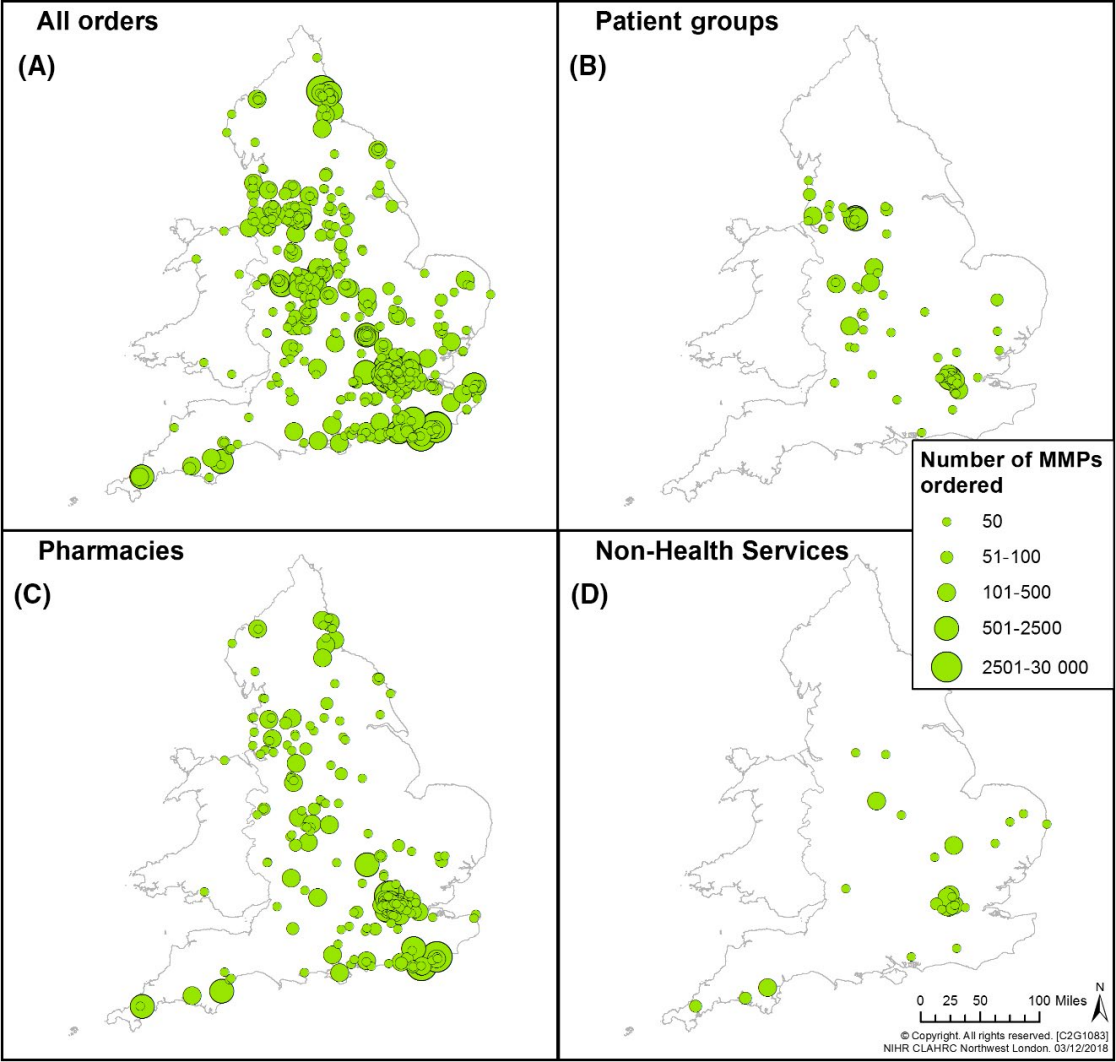
Diffusion of MMP in England and Wales by order type. Number of MMPs ordered by location in England and Wales between 5th April 2013 and 30th September 2017 for (A) all orders received, (B) orders from patient groups, (C) orders from pharmacies and d) orders from non‐health services (this includes third sector organisations, care centres, police services, universities)

Figures 2 and 3 are maps showing the extent of the geographical diffusion of the MMP innovation. Figure 2 shows the diffusion of MMP in Northwest London and hospital sites where the innovation was developed, piloted and where its initial use was evaluated.

Figures 2 and 3 demonstrate that the MMP innovation diffused beyond the sites where it was initially used to support a service improvement project. The success of that project led to further support being made available by NIHR CLAHRC NWL to create an online MMP order service. This enabled anyone to order it.^34^ Pharmacists placed the largest number of orders. Patient groups placed orders, identifying themselves as patients, patient representatives and members of GP Patient Participation Groups (PPGs). In addition, we mapped orders from non‐health organizations, including third sector organizations, care centres, police services and universities. Patients who ordered MMP were involved in initiatives based in acute, primary and community settings. Other occupations of those placing orders were categorized: administrative worker; care centre worker; charity, community interest or third sector worker; clinician (secondary care); GP or primary care practice staff; health‐care assistant; health service commissioning and related occupations; nurse; occupational therapist; physiotherapist; research.

### 3.2 Launch of MMP outside Northwest London

During 2012‐13, a steering group convened to “develop and implement a strategy for the roll‐out of ‘My Medication Passport’ across Northwest London.” In this same period, it became apparent that MMP was useful to a range of users and could be used outside of Northwest London. Key decisions taken by the steering group were to (a) secure funding for printing the MMP innovation, (b) develop an app and (c) set up a system to allow anyone to place an order via the NIHR CLAHRC NWL website. Among the key actions of that group was to effectively communicate about the innovation as widely as possible utilizing their resources and networks. Progress was discussed during monthly meetings attended by a multidisciplinary group which had 21 members: 2 patients/carers, 2 representatives from third sector organizations which had a patient/carer focus, 13 health‐care professionals including 4 pharmacists, 1 manager, 1 statistician, 1 evaluation lead for the pilot of MMP in Northwest London and 1 business consultant. A majority of the steering group had been involved in the development and/or the use of the MMP innovation in Northwest London. As members of the steering group, patients and carers were co‐producers of a strategic communications plan to help to disseminate MMP. Co‐production of the plan required all members of the group to suggest appropriate audiences. Patients and carers added community and third sector audiences and made initial contact with them to ask them to consider making MMP available via their own organizations and networks. These included national and local organizations that had regular contact with patients, older people, homeless people, library users, people who live with learning disabilities. They gave presentations about their experience of using MMP, the value it could provide for others and how to use it. They kept a record of the events that they and other steering group members attended which were linked to the strategy (n = 74) and they presented progress reports to the steering group.

Many, but not all the events were held in Northwest London. Some had regional, national and international audiences. The events were often led by patients alongside pharmacists and other health‐care professionals. Additional patients and carers helped facilitate the events. The exact numbers of additional patients and carers who were involved are not clear from available data. Two examples of how patient's roles developed and how the partnership processes between patients and health‐care professionals contributed to the diffusion are summarized below.

### 3.3 Professional networks

A key principle of the steering group's strategic communication plan was to raise awareness of MMP and encourage community and hospital‐based pharmacists to support it. Pharmacists who were members of the steering group were based in hospitals in Northwest London. They were able to use established hospital‐based networks to further disseminate the MMP. However, they had to establish contact with community‐based networks. Action taken was multifaceted. Pharmacists and patients worked together to co‐produce posters which were presented at in a variety of settings, in hospitals, local and national conferences and meetings. Pharmacists, carers and patients co‐created training materials containing advice on how MMP could be used. These were used in workshops and appeared on websites and in newsletters.^35^ The content of face‐to‐face training was co‐developed and delivered by pharmacists and patients. Patients volunteered in high street chemists, raising awareness about MMP. New relationships were built between pharmacists who were members of the steering group and other pharmacists, and between patients and pharmacists, and advocacy opportunities taken advantage of. Orders for MMP started to be placed. Figure 2 demonstrates the growth in orders placed for MMP in Northwest London from 2013. Figure 3 shows the growth in orders across the UK, and that the largest number of orders was placed by pharmacists.

### 3.4 Role of a carer:  linking to new networks

A member of the steering group, a carer, who is a co‐author of this paper, had the unique experience of caring for her son who had learning disabilities. He took multiple medicines and experienced fits related to his health condition. Her experience was that MMP had been useful to her and her son outside, as well as in health‐care settings. Their experience was that people who live with learning disabilities and who take multiple medicines can be “stopped and searched” for exhibiting unusual behaviour. This might be due to a health condition or medicine, and this could be misunderstood. Their experience of living with learning disabilities had brought them into close working contact with people who had mental health issues and who were sometimes homeless. As part of the steering group, she brokered the first of several meetings with the police force about MMP, and those meetings were attended by herself, her son and other steering group members. The aim was to introduce the police force to MMP and encourage their recognition of behaviour and medicines often related to people who have learning disabilities or mental health needs and/or who were homeless. A series of training workshops were agreed and delivered. Orders can be traced from these contacts and are mapped as part of those placed by non‐health services as shown in Figure 3.

### 3.5 Vignette 1. Patient participation group—GP practice and local community

This is a summary of a direct transcription from the Chair of a Patient Participation Group (PPG) for a medical centre in the North of England, who had heard about MMP from a patient network.

The GP surgery that the PPG supported had recently made patient access to their medical records freely available. It was found that not all patients wanted to access their records online, and not all had computers or access to the Internet. The Chair heard about MMP, and on consulting with GPs, and the PPG, it was decided that MMP could help support the needs of patients who preferred to carry information with them to health‐care appointments, or to show a friend or neighbour who would potentially support them if needed. She observed:

The PPG and GPs are keen to provide the best service for patients who can't or don't want to use online services, and I thought the MMP was a great solution.

I sent off for some passports and we started asking patients what they thought about them. Everybody thought they were great and the typical response was “Can I have another for my mum, husband, sister?”

The PPG worked with doctors and staff at the medical centres and it was agreed to offer patient's MMP to enable them to keep their own records, check these with their online record if they wanted to, and to use it as they wished.

The pharmacy based at the medical centre puts MMP into the prescription bags for patients with multiple medicines. Iterative feedback showed that patients and carers valued MMP and had ideas about other potential users with a known need for it. One carer said that when he took his wife in an ambulance, he took MMP with him and gave it to the consultant, who had welcomed it and said that “every patient should have one.”

In planning how best to support patients in the local area, the PPG considered patients they realized might benefit most from using the innovation and planned some outreach activities designed to bring MMP to their attention. For example, they supported the establishment of the use of the innovation in the local nursing home and via a local retirement housing scheme. A volunteer driver for nursing home resident's transport reported that he had been aware and worried that patients had no information with them about their medicines and no carer to listen or be their advocate during emergency and/or regular health‐care appointments. He became an advocate of the innovation and reported that patients used it.

The PPG made the innovation available to local community groups and they set up a stall in the local shopping centre. They had members who made it available to the local over 60s choir and facilitated their use of it.

The PPG was active in national networks and promoted the use of the innovation at the National Association for Patient Participation 2016 Annual Conference. They gave a presentation about how they had used it:

I spoke about MMP at the National Association for Patient Participation Annual conference in June 2016. We were presented with the award for “Most outstanding Patient Group in the UK!”

Other groups who had been at that conference have since been in touch with the medical centre and taken up the idea, including the local college who decided to use the innovation on their courses for people with learning difficulties.

### 3.6 Vignette 2. Advocates for patients who live with learning disabilities

This vignette is based on publications highlighting the role and experiences that one person who described himself as “carer, parent, patient and health‐care professional (pharmacist)” and the influence he had on others. He was an advocate for the use of the innovation and he published a case study about his own use of MMP to support the care of his son who has multiple disabilities including a learning disability.^27^

By publishing the case study and outlining the context in which the innovation was useful and through the publication of an additional article written for a broader audience with learning disabilities,^36^ at least two different sorts of peer community were influenced via peer and third sector networks.

First, professional networking between the author of the publication and colleagues within his own workplace influenced pharmacists and paediatric staff. Second, peer‐peer influence engaged paediatric services for patients with learning disabilities in a London hospital where MMP was extensively used. A poster about that service's use of the innovation was shown at a conference attended by peers in 2016.^37^ Third,  through personal experience of a third sector advocacy and support network, the author of the papers referred to above communicated effectively with the wider learning disability community. This was, demonstrated by a nationally recognized organization which endorsed the use of the innovation and made it available via their website to its users.^38^

### 3.7 Vignette 3. Promotion of MMP across social and professional networks

This vignette is a compilation of some brief examples of how the MMP innovation diffused though the networks of patients, carers and health‐care professionals.

A sports club for older people advocated the use of and disseminated MMP to its members after a man, one of its players, collapsed whilst playing cricket and required hospitalization. Friends of the man knew that he took multiple medicines but not which ones. They quickly became aware of how difficult it was for a hospital to secure any information that could confirm which medicines the man took, as both GP and community pharmacists were not open, and they recognized that the man lacked close family or friends who might have been able to help.

A local pharmacy group invited pharmacists who had experience of using MMP to their network events to explain how and why MMP had been developed, and to raise awareness about the potential benefits to pharmacists and to patients of using it. Several similar events led to training workshops being organized for pharmacists, and a training package aimed at their use of the innovation was developed and used.

A further example of the MMP innovation diffusion occurred between a hospital and patients who participated in a local PPG. Patients and carers used the innovation in the community. They discussed its potential to be of use to patients who used the local hospital pharmacy. That pharmacy went on to regularly order the innovation and to offer it to patients who took multiple medicines.

A health service network of staff and patients, and a community interest company that supports the diffusion of good practice relating to prescribing and supported NHS commissioners highlighted the MMP innovation in publicity and on their websites. They advocated the use of the innovation by patients, pharmacists and other prescribers to aid good practice.^39^, ^40^

## 4 DISCUSSION AND CONCLUSIONS

The authors' reflections on the findings are that patients played important roles in the diffusion of innovations shaping health service organization and delivery. These were as follows: advocate; relationship builder; relationship broker; planner; presenter; awareness raiser; trainer; networker; co‐producer. Patients identified and introduced potential audiences and users to MMP, using social, organizational, sectoral, lay and professional networks to do so. They organized a range of awareness‐raising and communication activities, monitored feedback, evaluated the impact and responded to new interest.

Sheard et al identified personal qualities of individual, award‐winning innovators who successfully influenced change in health services. Similar qualities can be seen among patients in the diffusion of MMP: personal determination; the ability to broker relationships; navigating organizational culture to good advantage; and the ability to use evidence to influence others.^23^ They used their inter‐personal and inter‐organizational networks, with some of the original innovators going on to become champions and leading efforts to achieve diffusion, for example within the police and prison service, in third sector organizations such as the sports club mentioned in vignette 3, and via ethnic minority health networks, community‐based groups as well as in libraries.^24^ The roles that patients and carers played, and their personal qualities were not unique to them. However, we draw attention to this because the roles and qualities are under‐recognized and overlooked in the literature as being linked to patients who influence change. In addition, the personal quality of empathy drove patients and carers to become advocates for others, influencing the use and diffusion of MMP.

Decisions about how to make the innovation available outside the setting and context in which it had been developed required strategic planning and corresponding action. Patients contributed to plans which they co‐created with others. The roles they played drew on their lived experience as patients and from other aspects of their lives as workers and carers; their insights, skills and personal qualities. They contributed to planning events to publicize the innovation: identified target audiences and potential users of it; created publicity material; developed key messages targeted to different audiences; decided target audiences and how to raise awareness among them; built relationships with them and supported the innovation's initial use in different settings. The timing, the resources required to support the activities and the necessary logistics to operationalize them were planned by patients for some audiences and by health‐care professionals for others. Patients were planners bringing communicators and networks together (vignettes 1, 2 and 3 and Tables 2, 3, 4). Patients, co‐planned and then, often but not always alongside pharmacists and other health‐care professionals, trained health‐ and non‐health‐care professional groups such as pharmacists, police and advocates for vulnerable groups of people such as those who are homeless or who had learning difficulties. We observed what Rogers had recognized as communication activities which were not linear and which, at various points, converged around sets of individuals working together^3^, ^4^ to use, and/or advocate or facilitate the use of the MMP.

All examples required experimentation, collaboration and trust between patients, carers and health‐care professionals in a common endeavour to spread knowledge about and encourage the use of the innovation. These factors underpinned new ways of working such as co‐production, an approach described by Green et al in the context of health service improvement projects.^10^ Filipe et al considered the concept of co‐production recognizing that “such a process involves dialogue and recognition of each other's capabilities and knowledge,” and this type of dialogue and recognition was evident in this study.^41^ Learning was shared between patients and health‐care professionals. This echoes deBronkart, who observed that attitudes towards knowledge and skills evolve and change when patients and health‐care professionals work together in mutual recognition that each can utilize their own experience and knowledge to improve care and support each other's learning.^20^

Strategic use of networks by patients points to several roles that can be played by patients and carers. In vignette 1, the PPG brought together local patients, carers and organizations, raised awareness and supported the use of MMP locally. They presented their experience at a national network conference influencing and supporting other PPGs. Parents and advocates for the innovation described in vignette 2 made connections between their own life‐experiences and those of others with whom they empathized. They influenced the strategy developed by the paediatric department of a hospital. In vignette 3, the coach of the sports club acted strategically, making the innovation available to all members of the club who took medicines, and the pharmacist who was also a carer advocated for disabled people and influenced awareness about MMP via a national third sector network. At the heart of these actions can be seen an ability to act strategically, use networks and the motivation to increase self‐care, self‐advocacy and patient safety.

Patients involved in the NIHR CLAHRC NWL steering group described in this case study had professional support from clinicians, patient and public involvement specialists and managers working in health services and third sector organizations. This necessary and supportive environment helped to enable the diffusion of the MMP innovation and effectively realize the value of patient involvement. The need for this support has been noted previously.^29^, ^30^, ^31^ Patients and carers described in this study had the benefit of organizational infrastructure, enabling them to co‐design, plan and work together with health‐care professionals on a variety of activities which helped diffuse the MMP innovation. The partnership between patients and health‐care professionals was critical to the diffusion of the innovation. It would not have occurred without patients, but patients could not have achieved it alone. Unlike the majority of patient innovators described in the study by Oliveria et al,^1^ patients in this study helped develop and diffuse an innovation which has been used to meet the needs of patients and carers in diverse health‐care and social settings. The Oliveria study and ours demonstrate that patients can innovate and create interventions likely to be of use to others and that professionals can see beyond the view that patients may be simply a source of knowledge on a condition or experience. Our results show that in addition patients can become change agents, helping put innovations into practice.

As opportunities to raise awareness and increase the use of the innovation snowballed, so MMP diffused. Without a driving strategy, in this case initiated by the improvement programme in Northwest London, and then the steering group which made MMP available outside of that project, the progression from innovation development to dissemination would not have been successful. Its subsequent diffusion is unlikely to have occurred in the multiple and complex settings that it did and without pharmacists engaging and ordering MMP the extent of diffusion is likely to have been far more limited. The MMP innovation can be understood as successfully diffused partly because it was adaptable to new settings and used by multiple types of user, having a “hard core,” the MMP itself, and a “soft periphery.”^2^ This soft periphery can be described in relation to MMP diffusion, pointing to the multiple new users and contexts outside of the discharge from hospital context that MMP was originally developed for.

The diffusion of the MMP was supported by a collaborative organizational culture underpinned by the encouragement of principles of co‐production^21^, ^32^ which set the MMP innovation on its first steps towards diffusion. Without the set‐up of the order service and app, supported by NIHR CLAHRC NWL,^34^ the innovation would not have been available and therefore not diffused. As Greenhalgh et al^5^ and Oliveira et al^1^ pointed out, infrastructure to support wide use of an innovation is an important element in the potential for its successful diffusion.

The patients involved in this study recognized that each potentially new setting they planned to introduce MMP in came with its own opportunities and challenges, including values, interests and power dependencies^2^ and that their role was to navigate those variables and build relationships.

As the MMP innovation diffused, communication roles played by patients brought MMP to the attention of a diverse range of audiences. To achieve this, patients demonstrated their use of multiple life‐experiences to inform opportunities and reach identified audiences. For example, the Chair of the PPG in vignette 1 was an advocate for the use of the innovation and brought it to the attention of a national support network of PPGs, explaining her use of it and how that could be adapted. Subsequently, she supported other PPGs that asked for more insight about how and when to use it, making the case for its use to support vulnerable groups of people.

Individual agency and collective action taken by patients and health‐care professionals shaped diffusion of MMP. Examples, of agency demonstrated through independent and collective communication and action presented in this study, have similarities to Filipe's account of co‐production as an experiment. She pointed to this as best “… seen as generative processes that are less about delivering predictable impacts and outputs and more about developing new communities, interactions, practices, and different modes of knowledge and value production.”^41^ Influential communication is important to achieving this. A key dynamic was the interaction and mutual support between patients and health‐care professionals. Patients built relationships within their existing personal, social and professional networks and collaborated with health‐care professionals in ways that they had not previously. They introduced health‐care professionals to networks; health‐care professionals introduced patients to theirs. Patients made new relationships with networks that they had not previously interacted with. For example, patients involved in communication events summarized in Table 3 and audiences summarized in Table 4 reported that they had not had any previous contact with homeless people, or with some faith groups before building relationships with them to offer, demonstrate and advocate the use of MMP. Opportunities opened up for patients and health‐care professionals to access previously untapped audiences.

Greenhalgh et al^5^ conceptualized the potential drivers of diffusion, key among which is communication. Their recognition that the boundaries between drivers are in most practical circumstances, fluid and have overlap, informed our analysis. What was observed was a non‐linear set of interactions during diffusion. Greenhalgh et al^5^ suggested that a question, which had been under‐researched, was “what mix of factors tend to produce adoptable innovations which are readily adaptable to new contexts?” One key factor in considering determinants of diffusion of MMP is the recognition and attribution of the multiple roles that patients and carers played alongside pharmacists and other health‐care professionals suggesting there is a blurring of boundaries between roles and this aligns with a principle of co‐production.^7^, ^42^, ^43^

This study demonstrated what can occur when patients innovate and are supported to develop their innovations for wider use by others. Infrastructural support from health‐care organizations and external funding to enable and make the MMP available underpinned its diffusion, and without this, diffusion was unlikely to have occurred. The study suggests that co‐working and co‐creation between patients, carers, pharmacists and other health‐care professionals in planning and progressing the use of the innovation was important, shaping the potential for patients to successfully play leading roles in MMP's diffusion. The enabling culture shaping the study was important. Reed et al^30^ point out, in their assessment of strategic principles, common challenges and simple rules that can guide health‐care improvements, that there is a need to embrace complexity, develop a holistic understanding of the strategic and practical possibilities and, in so doing, engage and empower all who have a role to play in delivering innovation. This study identified key roles played by patients in the diffusion of the MMP innovation: advocate; relationship builder; relationship broker; planner; presenter; awareness raiser; trainer; networker.

There are three main lessons from this study which may be useful for others interested in supporting patient roles to diffuse innovations. Firstly, we identify that patient's and carer's who are innovators, as well as those who are users of and advocates for an innovation, can act as effective leaders in disseminating and diffusing the innovation. Secondly, leadership qualities in patients and carers can be effectively utilized and nurtured when key gatekeepers (eg health‐care professionals) holding professional, financial and/or political power fully support co‐production and value patient and carer experience and knowledge. Thirdly, co‐producing a communications strategy can shape and validate roles for patients, carers and others to reach specific audiences. All of the above is more likely to succeed if the innovation has been evaluated before attempts to widely disseminate it and if the evaluation has demonstrated the acceptance of the innovation in practice by the intended users and audiences. These lessons are relevant to progressing other patient‐led ideas for innovative changes in relationship to health services and patient priorities in the UK and beyond.

## CONFLICT OF INTEREST

No known conflicts of interest.

## AUTHORS' CONTRIBUTIONS

Susan Barber: substantial contributions to conception and design, acquisition of data, analysis and interpretation of data, involved in drafting and revising the manuscript to final draft in collaboration with co‐authors. Catherine French: substantial contributions to conception and design, involved in drafting and revising the manuscript. Rachel Matthews: substantial contributions  in drafting and revising the manuscript critically for important intellectual content. Derryn Lovett: acquisition of data, analysis and interpretation of data relating to the diffusion of the innovation as evidenced via the electronic orders system, mapping that to Figures 2‐4; review and contribution to manuscript drafts. Tom Rollinson: acquisition of data, analysis and interpretation of data relating to the diffusion of the innovation as evidenced via the vignettes presented; review and contribution to manuscript drafts. Fran Husson: acquisition of data, analysis and interpretation of data relating to the diffusion of the innovation as evidenced via Tables 2‐4 relating to the launch of MMP nationally 2013‐14; review of manuscript drafts. Margaret Turley: acquisition of data, analysis and interpretation of data relating to the diffusion of the innovation as evidenced via Tables 2‐4 relating to the launch of MMP nationally 2013‐14; review of manuscript drafts. Julie Reed: substantial contributions to conception and design, involved in drafting and revising the manuscript.
